# Editorial

**DOI:** 10.1007/s00591-021-00311-w

**Published:** 2021-10-15

**Authors:** Ilka Agricola, Eva Müller-Hill

**Affiliations:** 1 Philipps University of Marburg, Marburg, Germany; 2 University of Rostock, Rostock, Germany

Since 2020, the global COVID-19 pandemic has been threatening our health worldwide, and the drastic measures taken for slowing down its spread have severely impacted our lives. Within weeks, it became a major challenge to science for the next years, if not decades. Research activities in medicine, pharmaceutics, and chemistry were closely monitored by the interested public and received intensive media coverage.

The contribution that mathematics can make to a deeper understanding and possible containment of the pandemic is less visible, but by no means less important. Mathematical epidemiology and statistics provide decisive tools to describe and capture relevant data and parameters.

However, all predictions and assessments of the impact of interventions such as social distancing, face masks, or vaccination rely on highly non-trivial mathematical models. Such models are only accessible to a very limited number of members of society, and not in a way that makes the application of these models meaningfully discussable in a wider circle.

We have decided, as our small contribution to a deeper understanding of mathematical modelings of the situation, to start a series of articles in the “Mathematische Semesterberichte” dealing with various mathematically tangible aspects of the pandemic; the first two articles can be found in this issue. Our aim is to cover a large variety of topics in such a way that they will still be worth reading in a few years. We believe that it is the duty of the mathematical community to contribute to the broader discussion, and in particular to describe relevant mathematical findings in an understandable and accessible way.

Our special thanks go to our authors and their anonymous reviewers alike. They all took on the burden of working much faster than usually under the aggravated conditions of an ongoing pandemic. Without their efforts and dedication, our project would not have been possible.

October 2021, Marburg and Rostock, Ilka Agricola and Eva Müller-Hill

